# Pulsed electromagnetic fields (PEMF) as a valid tool in orthognathic surgery to reduce post-operative pain and swelling: a prospective study

**DOI:** 10.1007/s10006-024-01256-9

**Published:** 2024-05-03

**Authors:** Marco Friscia, Vincenzo Abbate, Gianluca Renato De Fazio, Lorenzo Sani, Raffaele Spinelli, Stefania Troise, Paola Bonavolontà, Umberto Committeri, Luigi Califano, Giovanni Dell’Aversana Orabona

**Affiliations:** Maxillofacial Surgery Unit, Department of Neurosciences, Reproductive and Odontostomatological Sciences, University Federico II, Via Pansini 5, Naples, 80131 Italy

**Keywords:** Orthognathic surgery, Pulsed Electromagnetic Field, PEMF, Post-surgical swelling, Post-surgical pain

## Abstract

**Purpose:**

PEMF (pulsed electromagnetic fields) founds application in several medical fields to accelerate bone wounds healing and to reduce inflammation. The aim of our study was to evaluate the effectiveness of PEMF in reducing postoperative swelling and pain in patients undergoing orthognathic surgery.

**Methods:**

A prospective observational monocentric study was conducted on a sample of 30 patients undergone to orthognathic surgery in Maxillofacial Surgery Unit of University of Naples Federico II. The patients who followed these inclusion criteria were enrolled in the study: age ≥ 18 years, Class III malocclusion, Surgical procedure of Le Fort I osteotomy + Bilateral Sagittal Split Osteotomy (BSSO), Written informed consent. Patients were divided into two groups: Group SD) postoperative standard treatment with medical therapy and cryotherapy, Group SD + PEMF) postoperative standard therapy + PEMF. Each patient underwent a 3D facial scan, at one (1d) and four (4d) days after surgery to compare the swelling reduction. The pain score was assessed through VAS score and analgesics administration amount.

**Results:**

In SD + PEMF group, the facial volume reduction between 1d and 4d scan was on average 56.2 ml (6.23%), while in SD group, it was 23.6 ml (2.63%). The difference between the two groups was 3.6% (*p* = 0.0168). VAS pain values were significantly higher in SD group compared to SD + PEMF group in the second day after surgery (*P* = 0.021) and in the total 4 days (*P* = 0.008).

**Conclusions:**

Our data suggest that PEMF is valid tool to promote faster postoperative swelling and pain reduction in patients undergoing orthognathic surgery.

## Introduction

Orthognathic surgery is based on osteotomies aimed at correcting dentofacial deformities. Although in the recent years, minimally invasive approaches have been employed to minimize postoperative swelling and patient hospitalization [1], the most relevant complications of this surgery are related to the post-operative edema, pain and patient’s discomfort. To manage these complications, corticosteroid and non-steroidal anti-inflammatory drugs have been introduced in post-operative therapeutic protocols considering their effectiveness in swelling reduction [2], as well as ice packs for the vasoconstrictor action. In addition to conventional therapy, other treatments have been studied and approved [3].

The Hilotherm system [(Medival S.r.l. - Padova (Italy)] is a postoperative treatment, effective in reducing facial swelling in patients undergoing orthognathic surgery for dentofacial deformities. Through ice mask application in the lower face, the Hilotherapy induces vasoconstriction with a decrease in local tissue perfusion preventing the post-operative swelling [4, 5]. 

Among other treatments aimed at a rapid and optimal post-operative recovery, in recent years, PEMF (pulsed electromagnetic fields) has found application in several medical fields. PEMF uses low-frequency electromagnetic waves that can active signaling cells process and then stimulate cells regeneration. Cadossi et al. affirm that PEMF can promote the synthesis of skeletal extracellular matrix, accelerating the process of bone callus formation and, thus, the fusion of the bone stumps [6]. As reported in 2018 by Yuan et al., biophysical stimulation could be a non-invasive and safe physical therapy strategy to accelerate bone repair [7]. Therefore, PEMF is considered clinical routine in orthopedic surgery, and its effectiveness in reducing the bone union time and in increasing fracture healing, have been reported both in tibial fractures [8] and in femoral neck fractures [9]. This mechanism is based on the power of PEMF to promote osteogenesis and osseointegration of titanium implants/plates, without causing their movements, through a β-catenin signaling-associated process [10]. Moreover, PEMF has found application in oncology, due to the antineoplastic action [11] and in plastic surgery, due to the power to accelerate wounds healing [12, 13]. 

Electromagnetic fields not only stimulate bone healing and ossification, but are also able to reduce post-operative inflammation by the modulation of the immune and endocrine system. In fact, Electromagnetic fields act on the cell membranes by modifying ions transport, inducing macrophages production and InterLeukin-B decrease, with a final anti-inflammatory effect [14, 15]. 

Starting from these assumptions, the Authors of this paper supposed that PEMF could play a role after orthognathic surgery, to reduce post-operative inflammation, in terms of swelling and pain. Thus, the aim of this study was to investigate the effectiveness of PEMF in reducing post-operative inflammation, measuring the facial volume in ml and the administration of analgesics in patients undergoing orthognathic surgery.

## Materials and methods

### Study design and participants

A prospective observational clinical study was conducted from November 2021 to December 2022. All the recruited patients were hospitalized for dentofacial deformities and underwent orthognathic surgery. The inclusion criteria were:

- Patients aged 18 or over;
- Class III malocclusion;
- Surgical procedure: Le Fort I osteotomy + Bilateral Sagittal Split Osteotomy (BSSO).
- Written consent obtained in accordance with procedures defined and approved by the ethics committee following the registry plan (RP).

The exclusion criteria were: patients under 18 years old; patients with cleft of lip and palate; patients with bone metabolic diseases; patients already included in studies about the use of other treatments (devices or drugs) that may affect the outcome; other types of malocclusions or surgical procedures; failure of the acquisition of three-Dimensional (3D) facial scans; paracetamol allergy.

The ethical approval was obtained from the by the Ethics Committee of Biomedical Sciences of the “Federico II” University of Naples with the protocol number (392/21). The study was conducted in accordance with the Declaration of Helsinki.

All the patients who met the inclusion criteria were enrolled in the study and were divided into two groups: the first group (Standard Group - SD) included patients treated after surgery with glucocorticoids and Hilotherapy face mask; the second group (SD + PEMF) included patients treated after surgery with the standard therapy and PEMF. Assignment to one of the two groups depended on the device availability and on patient’s acceptance of the procedure. All surgical procedures were performed by the same operating team.

Both groups received the same postoperative medical therapy based on ceftriaxone 2 gr i.v. intraoperative and for 3 days after surgery, betamethasone 8 mg i.v. for 3 days and then gradually reduced over the next 6 days, gastric protection and analgesic therapy with ondansetron 8 mg/4mL + ketorolac tromethamine 60 mg/2mL + tramadol 200 mg/2mL through the elastomeric pump for the first 24 h.

All patients received the same amount of postoperative intravenous hydration (1500 cc of fluids for the first 3 days after surgery) and the same post-operative hospital care. All patients in both groups were instructed on face care and oral hygiene measures, including the use of 0.2% chlorhexidine digluconate mouthwash at least twice daily.

**Fig. 1:**
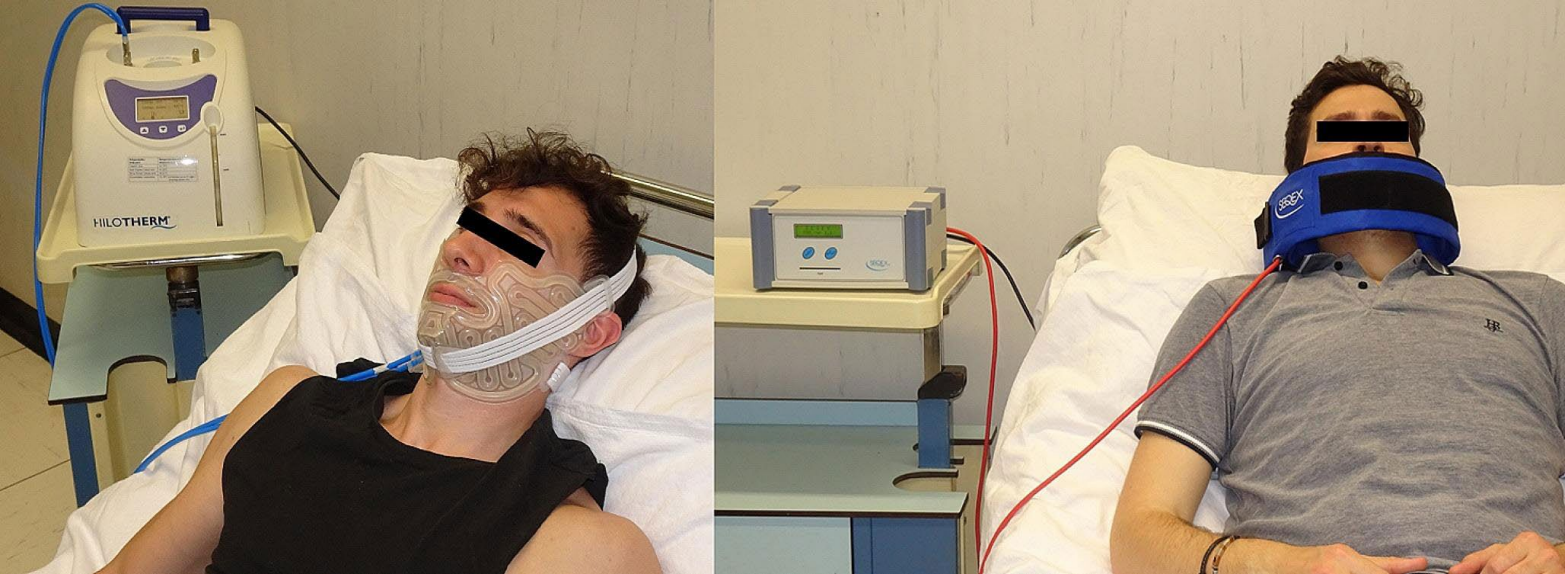
Hilotherapy applied facemask on the left and PEMF applied facemask on the right

Every osteotomy was performed by using piezosurgery (Piezosurgery Plus, Mectron s.p.a. 2014,) to reduce the thermal damage. Both groups of patients received cryotherapy through the facial mask of the hilotherapy system, with a set temperature of 11° C. The facial mask was placed inside the operating theater immediately after surgery and kept on for at least 24 h. It was periodically removed to allow patients to their personal hygiene and to feed themselves. (Fig. 1a)

The SD + PEMF group received PEMF through the electro-medical device SEQEX® FAM (S.I.S.T.E.M.I. srl, Italy) following a protocol drawn up by our Unit according to the scientific literature [7, 16] and to the indications of the technical suppliers. Our protocol has been codified to maximize the swelling prevention efficacy by the emission of low-frequency waves treatment.

SEQEX® FAM is a class IIa medical device, following the essential requirements of the current European directive 93/42 concerning medical devices. The device used in the study consisted in a control console, a power cable and a connector to a specific designed mat, called Anatomical Applicator. The application of magnetic fields modulated in intensity, and frequency and waveform, takes place with the aid of transducers contained within the mat connected to a control console. (Fig. 1b)

The electromagnetic therapy was applied 2 times per day (36 min each), alternately with Hilotherm system. This protocol was performed from 4 h after surgery until the 4th day postop, before discharging the patient from the hospital.

### Data sources and collection

Each Patient underwent a 3D facial scan the day after surgery (1d scan) and 4 days after surgery (4d scan), usually the last day in hospital, to obtain a facial volume in milliliters.

**Fig. 2:**
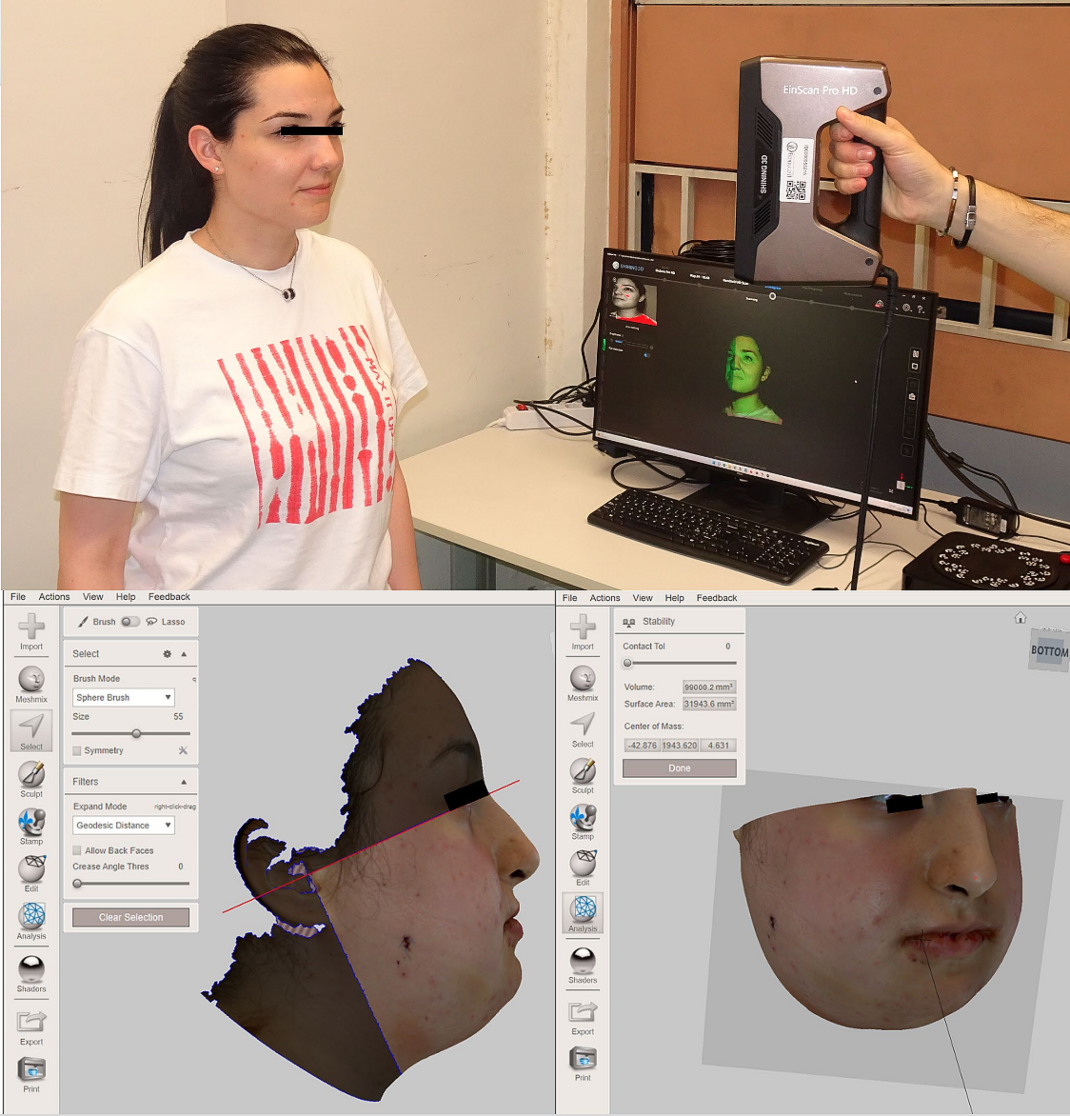
Upward the Face Scanning with Shining 3d Ein Scan Pro HD; below the Facial scans cutting on Meshmixer software based on two lines: the first passing between tragus and nasion; the second traced perpendicular to the first; the final scan was used to assess the reduction of volume between the first and the fourth post-operative day

Facial scans were acquired by the Shining 3d Ein Scan Pro High Definition (Hangzhou, China, 2019) which has a proven accuracy of 0.045 mm at a working distance of 510 mm. (Fig. 2a) The scans were all performed by the same operator, to reduce the risk of bias.

The scans were then uploaded to Meshmixer software ver. 3.5 (Autodesk, 2020). In order to compare 1d scan with 4d scan each mesh derived from the scans was cut by two lines: the first passing through tragus and nasion; the second was traced perpendicular to the first passing through the tragus to obtain comparable facial volumes. The volume in ml of each 3D mesh (1d and 4d) was calculated through the tool *mesh analysis*. (Fig. 2b, c)

For all patients, a visual analogue scale (VAS) was used to assess pain (0 mm: no pain at 100 mm: disabling pain) and the dose of analgesics administered was recorded. The measurements were carried out after the end of the elastomeric pump (in the second day after surgery), in the total of 4 days of hospitalization and at the seventh day, during the outpatient check-up. The analgesics dose was not considered at 7 days due to the impossibility to control the intake of the drug after discharge from hospital. Paracetamol 1000 mg was used as analgesic for all patients, to standardize the data.

### Variables

The primary predictor variable was the facial volume, measured in millimeters, and in particular the facial volume reduction between the 3D scan acquisitions on the first day after surgery and on fourth day after surgery. The secondary variables were the VAS SCORE and the analgesics amounts in mg. All the covariates, such as age, sex, smoke and BMI, were considered to avoid confounding factors.

### Statistical methods

The statistical analysis was performed with Statistical Package for Social Sciences, version 16.0, for Windows (SPSS, Chicago, IL, USA) and R (software), version 4.0.2, for Windows (R Foundation for Statistical Computing, Vienna, Austria). To perform the analysis of correlation between the primary variable and the covariates the Pearson’s regression coefficient (r) was used. For each categorical variable, a dummy variable was introduced, taking values of 0 (absence of the character) or 1 (presence of the character). In particular: - For *r* < 0.1 there is no discernible correlation between the two variables; - For 0.1 < *r* < 0.3 the correlation between the two variables is weak; - For 0.3 < *r* < 0.5 the correlation between the two variables is intermediate; - For *r* > 0.5 the correlation between the two variables is strong. After the analysis, the Pearson’s coefficient was standardized as *p*-value and a *p* < 0.05 was considered statistically significant for each correlation analysis.

To compare the facial volume reduction in the two groups, a data distribution test was performed. The data distribution was abnormal due to the small sample size; therefore, a non-parametric test was applied. Using the non-parametric Mann-Whitney U test (group comparison), the data were examined for signs of significant differences. A value of *p* < 0.05 was considered statistically significant.

To compare the VAS SCORE results and the administrated analgesics in the two groups, the statistical analysis was performed using non-parametric Mann-Whitney U test (group comparison); statistical significance was defined at *P* < 0.05.

## Results

### Participants and descriptive data

The sample consisted in 30 patients (11 male, 19 female), with a mean age of 25 years old, and an average BMI of 22,8. The SD group included 15 patients, 10 females and 5 males with a mean age of 25 years old and an average BMI of 22,4. The SD + PEMF group included 15 patients, 9 females and 6 males with a mean age of 23 years old and an average BMI of 23,2. Among all the patients, 9 were smokers (5 in the SD group and 4 in the SD + PEMF group); no one of the patients either was alcohol consumers or had any comorbidities.

### Outcome data and main results

None of the covariates was found to have a statistically significant impact on the primary variable (*p* > 0.05; *r* < 0.1)(Table 1).

**Table 1:** Sample features

	SD Group	SD + PEMF Group	Correlation to Volume reduction
Mean Age	25 years old	23 years old	*P* > 0.05
Sex M/F	5/10	6/9	*P* > 0.05
BMI	22,4	23,2	*P* > 0.05
Smoke	5	4	*P* > 0.05
Alcool	0	0	-
Comorbidities	0	0	-

In the SD + PEMF group, the facial volume reduction between the 1d scan and 4d scan was on average 56.2 ml (6.23% of the initial volume), while in the SD group, it was 23.6 ml (2.63% of the initial volume) (Table 2). The difference between the volumetric reductions of SD + PEMF and SD was 3.6%. These data show that SD + PEMF therapy resulted in 3.6% more de-swelling than SD therapy. The Mann-Whitney U Test showed a statistically significant variation (*p* < 0.05) between the two groups with a relevant decrease in the volumetric values in the SD + PEMF group. In particular, the U-value was 40, the critical value of U at *p* < 05 is 70; the Z-Score was − 3.14251. The *p*-value was 0.0168. Thus, the result was significant at *p* < 0.05.

**Table 2:** Volumetric values for each patient in the study groups

Patients nr	SD-Group				SD + PEMF-Group				*P*-value
	Vol. 1d (mL)	Vol. 4d (mL)	Δ	%	Vol. 1d (mL)	Vol. 4d (mL)	Δ	%	
1	857.40	822.8	34.6	4.20	891.4	796.6	94.8	10.63	
2	990.0	944.9	45.1	4.56	848.5	769.6	78.8	9.29	
3	900.7	889.8	10.9	1.21	1084.1	1052.5	31.6	2.92	
4	1089.0	1073.6	15.4	1.41	869.8	797.2	72.6	8.34	
5	884.5	874.2	10.3	1.18	814.9	760.2	54.8	6.72	
6	817.5	772.4	45.0	5.51	1145.8	1133.2	12.6	1.10	
7	884.8	861.3	23.6	2.67	1040.1	955.7	84.4	8.11	
8	932.5	919.2	13.3	1.42	837.1	791.8	45.3	5.41	
9	1055.7	1039.3	16.4	1.55	852.2	796.2	56.0	6.57	
10	731.3	710.6	20.7	2.84	1016.3	979.3	37.0	3.64	
11	976.4	947.8	28.6	2.93	988.5	915.6	72.9	7.37	
12	875.4	857.8	17.6	2.01	945.7	901.3	44.4	4.69	
13	1002.1	983.2	18.9	1,89	879.5	832.4	47.1	5.36	
14	988.2	961.4	26.8	2.71	1005.7	938.6	67.1	6.67	
15	895.6	864.9	30.7	3.43	857.6	801.7	55.9	6.52	
**Average value**	**941.3**	**917.7**	**23.6**	**2.63**	**936.2**	**883.3**	**56.2**	**6.23**	***0.0168***

VAS pain values were significantly higher (*P* < 0.05) in the group SD compared to group SD + PEMF in the second day after surgery (*P* = 0.021) and in the total 4 days (*P* = 0.008), but there was no significant difference (*P* > 0.05) on the day 7. (Table 3). The total dose (in mg) of administrated analgesics in the second day after surgery (*P* = 0.0064) and in the total 4 days (*P* = 0.0001) was significantly lower in SD + PEMF group than SD group (Table 4).

**Table 3:** VAS (visual analog scale) Score results in the two groups

VAS SCORE	SD Group Mean ± SD	SD + PEMF Group Mean ± SD	*P* Value
Second day	59.6 ± 12.2	41.6 ± 11.1	*P* = 0.021
Total 4 days	47.3 ± 8.2	28.2 ± 6.4	*P* = 0.008
Total 7 days	14.7 ± 5.3	10.3 ± 4.2	*P* = 0.123

**Table 4:** Distribution (in mg) of the analgesics administration in two groups

Analgesics Dose	SD Group Mean ± SD	SD + PEMF Group Mean ± SD	*P* Value
Second day	2272.73 ± 786.25	545.45 ± 687.55	*P* = 0.0064
Total 4 days	6769.23 ± 1235.17	2769.23 ± 725.01	*P* = 0.0001

## Discussion

In this study, the Authors investigated the role of PEMF as a support tool in reducing postoperative edema and pain after orthognathic surgery, calculating the facial volume difference in ml between the first and the fourth day after surgery and evaluating VAS score results for pain. The Authors’ hypothesis was based on recent studies that demonstrated the effectiveness of PEMF therapy in orthopedic surgery. This device is based on low frequency electromagnetic fields that induce the ions exchange through cell membranes, causing a depolarization of the cells. Usually, the physiological cells voltage is between − 20 and − 25 V, and when it varies, the cells begin to produce pain mediators and inflammatory cytokines, such as interleukin-1 beta (IL-1β) [17]. PEMF, by restoring the correct depolarization, interrupts the inflammation cascade; in particular, increasing the Calcium-ions flow, the electromagnetic fields block the IL-1β release and increase protein synthesis, oxygenation, ATP production by mitochondrial cell function, vasodilation and circulation, that are stimulated by the Ca^2+^ intracellular cascade [18, 19]. 

Moreover, PEMF accelerates osteogenic differentiation through the Ca2+/nitric oxide/ cGMP/protein kinase G pathway, that promotes osteoblast differentiation and maturation [20]. For these reasons, this method has been used for cells regeneration and bone wounds repair, in particular in case of nonunion fractures [21]. 

Considering that the post-operative pain and edema are a consequence of the activation of the same cell mediators on which PEMF can act [22], the Authors of this paper hypothesized that this method could be used to reduce these complications after orthognathic surgery. The underlying principle of this hypothesis was the possibility of PEMF to block the inflammation cascade and, consequently, the development of post-surgery edema and pain.

The effectiveness of this tool was evaluated comparing the facial volume in ml, acquired trough 3D facial scans performed the 1st and the 4th day after surgery, the day of discharge from hospital. The scans were all carried out by the same operator as well as the volumetric calculation, to avoid any bias in image acquisition or measurements.

The interesting evaluation was to analyze the decrease of the facial volume between the first and the fourth day after surgery in same patient: in this way all the variables related to BMI or to the individual healing times, were found to be negligible.

The results analysis showed a reduction of post-operative swelling in the SD + PEMF Group of 6.23% on average compared to a 2.63% reduction obtained in the SD group, showing satisfactory results of the applied protocol. Thus, regardless of the patients’ starting volumes recorded on the first day after surgery, the average Δ was higher in the group SD + PEMF, confirming that greater tissue remodeling was recorded in patients undergoing PEMF.

However, the Authors were questioned on how electromagnetic fields can reduce edema, although they stimulate vasodilation? The answer lies in some studies that, both in vitro and in vivo, have shown that PEMF increases blood flow to the healing tissues, limiting the extravasation of liquids [23]. In the case of orthognathic surgery, osteotomies represent the site where the tissue must heal, so PEMF increases blood flow to the bone, reducing extravasation of fluids into the surrounding soft tissues. The effectiveness of PEMF has already been showed in oral surgery, in particular in implantology [24], in mandibular traumatology [16] and in orthodontic treatment [25] but, to the best of our knowledge, this is the first articles aimed at evaluating quantitatively the swelling prevention efficacy of PEMF and the reduction of pain in patients treated by orthognathic surgery.

The clinical relevance of using PEMF after orthognathic surgery can be reducing the corticosteroids dosage limiting eventual adverse reactions, improving the psychological impact of surgery; fast recovery of normal feeding and swallowing, and accelerating patient’s return to daily social life. Nevertheless, this study has some limitations: monocentric survey; small sample of only 30 patients; short PEMF treatment lasting time (4 days). Multi-centers studies on larger sample sizes will be needed to verify our preliminary results. Moreover, no evaluations were carried out on the changes in blood inflammatory indices in the post-operative period and the effect of PEMF in the absence of corticosteroid intake was not evaluated. Therefore, these analyzes could be a starting point for interesting future research.

Our future perspective is to use PEMF for a longer post-operative time, to evaluate the effect of on bone healing at the level of osteotomies and to carry out an evaluation of the changes of the inflammatory blood biomarkers also without the administration of corticosteroid therapy.

## Conclusion

The encouraging results, obtained from our analysis, seem to suggest that PEMF is an effective tool to support standard therapy in reducing post-operative swelling and pain in patients undergoing orthognathic surgery. This device could be routinely used after orthognathic surgery to accelerate the post-surgical recovery, reducing the amount of administrated analgesics.

## Data Availability

No datasets were generated or analysed during the current study.

## Declarations

### Guidelines check list

The study complies with the STROBE checklist: case-control studies.
